# Correcting the Site Frequency Spectrum for Divergence-Based Ascertainment

**DOI:** 10.1371/journal.pone.0005152

**Published:** 2009-04-16

**Authors:** Andrew D. Kern

**Affiliations:** Department of Biological Sciences, Dartmouth College, Hanover, New Hampshire, United States of America; Washington University, United States of America

## Abstract

Comparative genomics based on sequenced referenced genomes is essential to hypothesis generation and testing within population genetics. However, selection of candidate regions for further study on the basis of elevated or depressed divergence between species leads to a divergence-based ascertainment bias in the site frequency spectrum within selected candidate loci. Here, a method to correct this problem is developed that obtains maximum-likelihood estimates of the unascertained allele frequency distribution using numerical optimization. I show how divergence-based ascertainment may mimic the effects of natural selection and offer correction formulae for performing proper estimation into the strength of selection in candidate regions in a maximum-likelihood setting.

## Introduction

The recent explosion in genome sequencing has proven to be an invaluable resource to evolutionary genetics. For the first time investigators are able to explore hypotheses about evolutionary processes that shape DNA at the level of whole genomes. In particular those genomic regions that have evolved either very slowly (e.g. [1]) or very quickly (e.g. [2]) among species are of considerable interest, as the evolution of these regions should be dominated by the deterministic forces of natural selection [3].

The population genetics approach of comparing patterns of genetic divergence among species to patterns of polymorphism within species provides a powerful tool towards unraveling the myriad of forces at work in genetic evolution. A typical approach is as follows: 1) pick candidate regions for study based on levels of divergence between reference genome sequences, and then 2) attempt to infer causes of evolution based on polymorphism data from those candidate regions. An example of the approach is the work of Drake et al. [4], wherein the authors attempted to understand whether conserved non-coding sequences (CNCs) in the human genome were selectively constrained or mutational cold-spots. As the allele frequency distribution from CNCs was shifted towards rare alleles relative to non-conserved sequences, the authors concluded that selection against deleterious mutations was occurring in CNCs, and thus such sequences were likely to be functional and not in fact mutationally cold.

**Figure 1:**
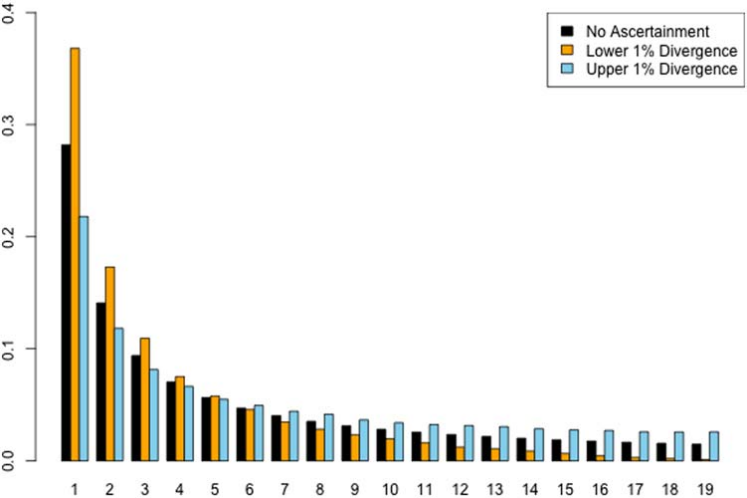
The effect of divergence-based ascertainment of the site frequency spectrum. Coalescent simulations were performed to generate 10^6^ unlinked genomic regions of 20 ingroup individuals and a single outgroup sequence, with a species divergence time of 5.0 4*N_e_* generations and a fixed value of 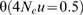 and 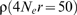. Levels of divergence were then calculated in these regions by selecting a single, random ingroup sequence and comparing it to the outgroup. From this comparison, those regions within the observed upper and lower 1% of divergence were retained. Shown are the unfolded site frequency spectra from the upper (blue) and lower (orange) 1% regions compared to the expected site frequency spectrum under the standard neutral model (black). Ascertainment based on low levels of divergence biases the recovered site frequency spectrum towards rare alleles, relative to the standard neutral model, whereas ascertainment based on elevated levels of divergence biases the site frequency spectrum towards intermediate and high frequency alleles.

The problem with this approach is that selection of candidate regions based on patterns of divergence between species creates an ascertainment bias with rather large effects on the expected site frequency spectrum (Fig. 1). Intuitively, this can be understood by considering that polymorphisms can often be confused with fixed differences between species when single, or few, sequence comparisons are used to measure divergence. In the case of genomic regions selected for conservation, divergence-based ascertainment biases against intermediate or high frequency derived alleles, as such alleles would likely show up in between species sequence comparisons as fixed differences, causing candidate regions to be rejected. Conversely, genomic regions identified as rapidly evolving, are expected to be enriched for high and intermediate frequency polymorphisms, as these are more likely to contribute to the signal of divergence (see [5]). It is also worth noting that this skew in the frequency spectrum will lead to an increase or decrease in the expected number of segregating sites recovered from divergence ascertained loci.

## Analysis

**Figure 2:**
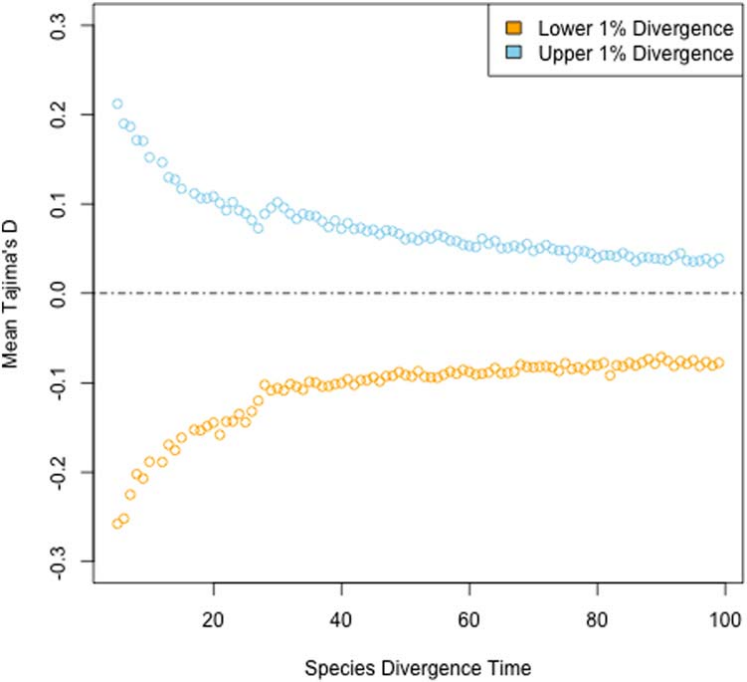
The effect of species divergence time on divergence-based ascertainment bias. Coalescent simulation as in Figure 1 were performed, however a species divergence time was varied between 5 and 100 4*N_e_* generations was used (see Figure 1 caption for simulation details). Shown are values of Tajima's *D* from the upper (blue) and lower (orange) 1% regions. A dotted line at *D* = 0 is shown for reference to the neutral expectation. The strength of a divergence-based ascertainment bias decreases as a function of species divergence time, but does very slowly such that an appreciable effect remains at species divergence times of 100.

As the divergence time between species increases, the expected ratio of fixed differences to segregating sites in a sample also increases. One should then expect the strength of a divergence-based ascertainment bias to decrease with increasing genetic distance between species used in the ascertainment phase. This decay in divergence-based ascertainment bias is shown in Figure 2, where coalescent simulations are performed as in Figure 1 but species divergence times vary. Tajima's *D* [6] is used here as a one dimensional proxy for the site frequency spectrum. Even at species divergence times as great as 100 (in units of 4*N_e_* generations) a substantial divergence-based ascertainment bias is apparent.

**Figure 3:**
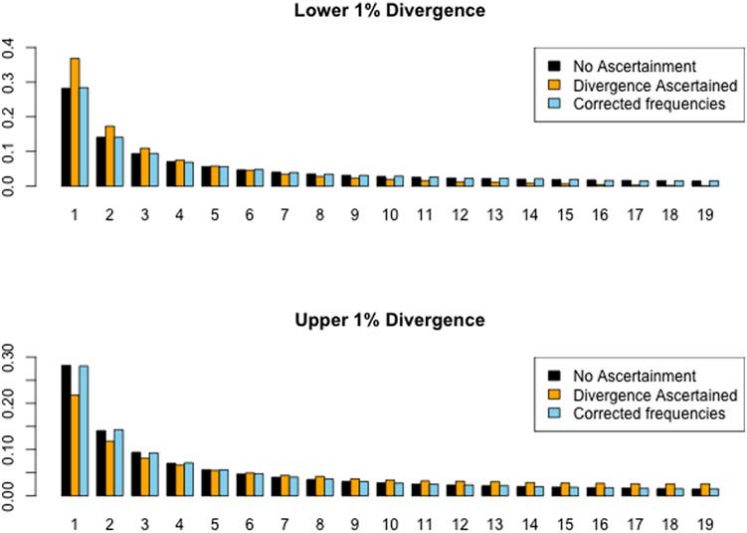
ML estimates of the site frequency spectrum from divergence ascertained data. Data used in the top and bottom panels correspond to the lower and upper 1% divergence ascertained data simulated via a coalescent method (see Figure 1 caption for details). Shown are the unfolded site frequency spectra from the divergence ascertained data (orange), ascertainment-corrected data (blue), and the expected standard neutral model spectrum (black).

To correct for this divergence-based ascertainment, we wish to obtain an estimate of the unascertained site frequency spectrum. Following the general framework of Nielsen et al. [7], let *p_j_* be the frequency of segregating sites with derived allele frequency *j* in a sample of *n* chromosomes that has undergone no ascertainment bias (1<*j*≤*n*−1). With a set of observed counts of allele frequencies in hand we aim to find the maximum-likelihood estimate of the site frequency spectrum, 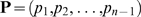. We assume that the presence or absence of the derived version of each segregating site in the original divergence comparison is known. The likelihood function for the site frequency spectrum, 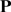 is then
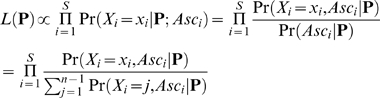
(1)where *S* is the number of segregating sites in the sample, *X_i_* is the derived allele frequency at the *i*th site including those in the reference genome used to ascertain the site, and *Asc_i_* is the notation for the ascertainment of a segregating site in the original divergence comparison. It is clear that 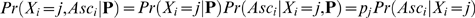 since ascertainment depends only on how many ancestral and derived actually occur at site i (Nielsen et al. [7]). Thus the crucial quantity for correcting divergence-based ascertainment is the probability of our ascertainment condition, which can be incorporated at a site by site basis to correct for arbitrary levels of divergence (see Figure 3). I assume that divergence ascertainment was performed in a subsample, size *d*, of our final sample, size *n*, thus the probability of ascertainment is
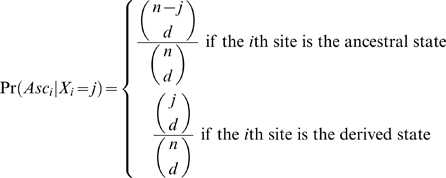



In most cases, for example that of Drake et al. [4], *d* = 1, corresponding to ascertaining divergence from a single genomic reference sequence. If *d*>1, divergence is assumed to be measured by the number of fixed differences (see [8] for a cogent treatment of this statistic).

With this probability in hand, one can then estimate 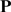 via numerical optimization of the likelihood function (Equation 1). In the present case optimization was performed by using a version of the Broyden-Fletcher-Goldfarb-Shanno (BFGS) algorithm [9] however any standard optimization scheme will work. Figure 3 shows the results of maximum-likelihood estimation of the site frequency spectrum from the simulated data presented in Figure 1 (source code to perform this correction is available upon request). As can be noted visually, very accurate estimates of the unascertained allele frequency distribution are recovered from the divergence ascertained data. It is important to note that unlike in Nielsen et al. [7] where the authors aim to find the “true” site frequency spectrum, we already know the site frequency spectrum of our region(s) and instead we aim to correct for poor ascertainment in which polymorphisms were mistaken for fixed differences in deciding which regions of the genome to study.

Often estimates of evolutionary parameters are of central interest to the investigator. In particular the strength of selection acting on a region may be an important quantity to examine (e.g. [10]). The framework presented here is easily incorporated into such estimation. One can describe the probability of a derived allele segregating in a sample at frequency *X_i_* as a function of its selection coefficient 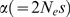, and thus to estimate the strength of selection acting on a locus with observed site frequency spectrum 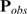 via maximum-likelihood (see [11]–[13]). Generically, the likelihood function takes the form
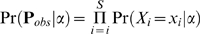
(2)(see [12], [13] for 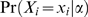). Using Bayes rule, and noting that the probability of divergence ascertainment is conditionally independent of α given *X_i_* = *x_i_*, i.e. 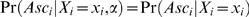, we can then write down the divergence ascertainment corrected version of Equation 1:
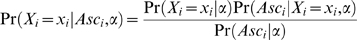
where the denominator is simply the normalization factor
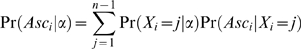



The divergence ascertainment corrected likelihood function can then be written as
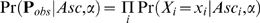
(3)


**Figure 4:**
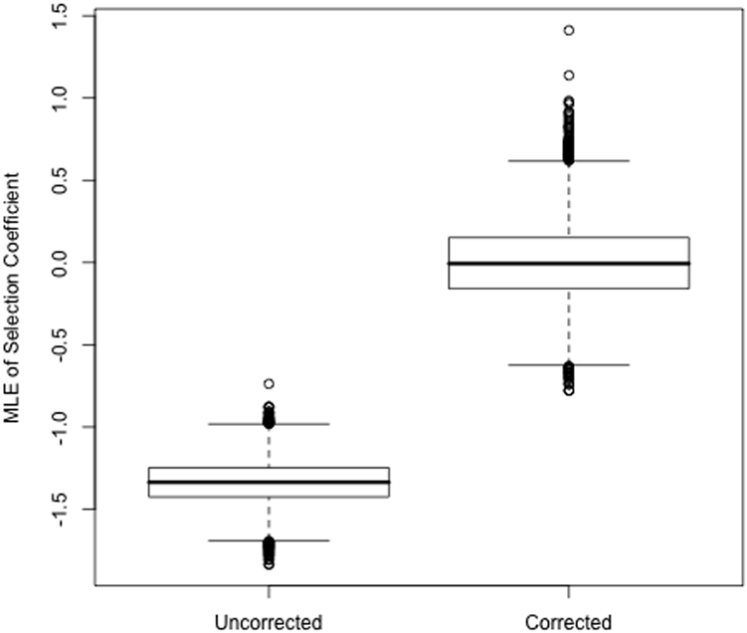
ML estimates of the strength of selection with and without divergence ascertainment correction for lower 1% data. The strength of selection, α, was estimated from those loci identified as belonging to the lower 1% divergence group from the 10^6^ simulated regions (see Figure 1 caption for details). The left box shows MLEs using an estimation routine which does not account for divergence-based ascertainment (uncorrected), the right box shows MLEs from the same data but estimated in a fashion which accounts for ascertainment (corrected). Note that uncorrected estimates show spurious evidence for negative selection even though the data were generated from a neutral model.

Figure 4 shows the effect of divergence-based ascertainment on ML estimates from simulated loci selected for depressed levels of divergence. The mean from uncorrected estimates (i.e. Equation 2) is α̂ = −1.34, indicating evidence of weak negative selection, even though these data have been generated under a standard neutral model. MLEs of α using the divergence-based ascertainment corrected likelihood function (Equation 3), restores the expected value to approximately zero (mean α̂ = 0.001). This ascertainment corrected version of the likelihood function is also useful in a Bayesian setting, for an example see Katzman et al. [14], where it was used in a Bayesian Hierarchical model for estimating distributions of selection coefficients from divergence ascertained data.

**Figure 5:**
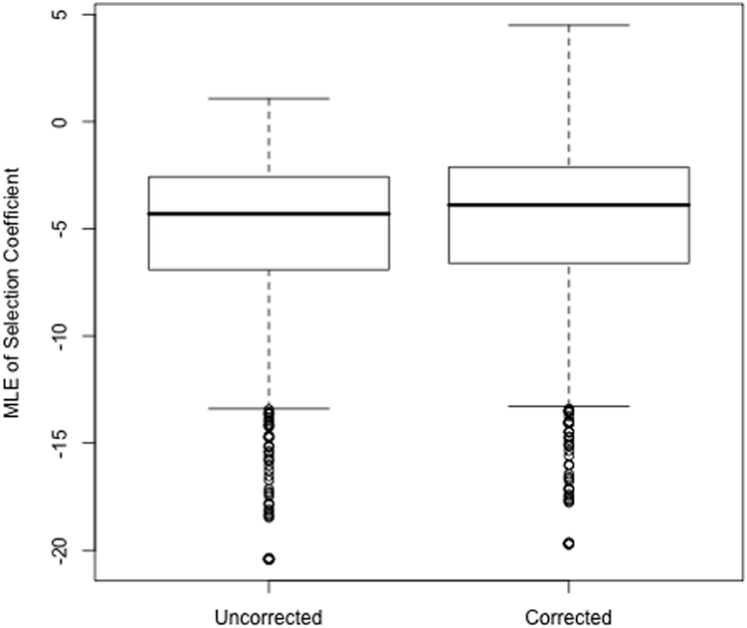
ML estimates of the strength of selection from simulations with selection with and without divergence ascertainment correction for lower 1% data. The strength of selection, α, was estimated from those loci identified as belonging to the lower 1% divergence group from the 10^6^ regions simulated from a deleterious alleles model with α = −5.0. Simulations were performed using a model closely related to Gillespie's exponential shift model [15], but rather than a distribution of selection coefficients only a single coefficient is assigned to new mutations (simulation details can be found in Kern et al. [16]). This method uses a time forward population genetic simulation approach. Population size for these simulations is 10^5^, all other parameters are identical to those in Figure 1. The left box shows MLEs using an estimation routine which does not account for divergence-based ascertainment (uncorrected), the right box shows MLEs from the same data but estimated in a fashion which accounts for ascertainment (corrected). As in the neutral setting, correction restores estimates close to their true value and evidence for negative selection once corrected for ascertainment can be thought of as conservative.

One concern with our ascertainment correction is that we might mask the “true” signature of selection acting at a locus. Figure 5 shows the effect of our correction on ML estimates of selection coefficients from loci simulated under a deleterious alleles model (i.e. negative selection; see figure caption for details) that again have been selected on the basis of depressed divergence. Of particular concern is the case of weak negative selection, thus simulations were performed where new alleles were assigned a negative selection coefficient with the strength of selection α = −5.0. Under this model, both the uncorrected and the corrected estimates were relatively close to the true selection coefficient (uncorrected mean α̂ = −5.36, corrected mean α̂ = −4.91), however the ascertainment correction does seem to improve estimates. Evidence of selection when estimates have been corrected for divergence ascertainment can thus be considered conservative.

## Conclusions

Reference genome sequences are an invaluable resource, however their utility in identifying candidate regions for further study presents the pitfall of divergence-based ascertainment biases in population genetics investigation. Divergence-based ascertainment bias calls into question the validity of earlier studies of the site frequency spectrum from regions which have been selected based on their evolutionary rate between species but have ignored this source of error. In this study I offer simple corrections for those biases which may be used to estimate the unascertained site frequency spectrum of divergence ascertained data as well as for estimation of evolutionary parameters when the sequence used for ascertainment is included in the population sample. In particular, accounting for ascertainment when estimating selection coefficients is imperative when loci have been selected on the basis of divergence between species.
